# NORE1A induction by membrane-bound CD40L (mCD40L) contributes to CD40L-induced cell death and G1 growth arrest in p21-mediated mechanism

**DOI:** 10.1038/cddis.2016.52

**Published:** 2016-03-17

**Authors:** T Elmetwali, A Salman, D H Palmer

**Affiliations:** 1 Department of Molecular and Clinical Cancer Medicine, Institute of Translational Medicine, University of Liverpool, Daulby Street, Liverpool L69 3GA, UK

## Abstract

Membrane-bound CD40L (mCD40L) but not soluble CD40L (sCD40L) has been implicated in direct cell death induction and apoptosis in CD40-expressing carcinomas. In this study, we show that mCD40L but not sCD40L induces NORE1A/Rassf5 expression in an NF*κ*B-dependant mechanism. NORE1A expression appeared to contribute to mCD40L-induced cell death and enhance cell transition from G1 to S phase of the cell cycle in a p21-dependent mechanism. The upregulation of p21 protein was attributed to NORE1A expression, since NORE1A inhibition resulted in p21 downregulation. p21 upregulation was concomitant with lower p53 expression in the cytoplasmic fraction with no detectable increase at the nuclear p53 level. Moreover, mCD40L-induced cell death mediated by NORE1A expression appeared to be independent of mCD40L-induced cell death mediated by sustained JNK activation since NORE1A inhibition did not affect JNK phosphorylation and vice versa. The presented data allow better understanding of the mechanism by which mCD40L induces cell death which could be exploited in the clinical development of CD40-targeted anti-cancer therapies.

We, and others, have demonstrated that the outcome of CD40 receptor activation by its ligand CD40L/CD154 in carcinomas predominantly relies on the way the ligand is presented to the receptor at the cell surface, where membrane-bound CD40L (mCD40L) but not soluble CD40L (sCD40L) induces cell growth arrest and apoptosis. mCD40L-induced cell death occurs through a mechanism that involves the downregulation of pro-survival signals, mainly the PI3K/AKT pathway and the sustained activation of the pro-apoptotic JNK pathway, leading to caspase activation and subsequently apoptosis.^1, 2^ However, the role of CD40L in cell cycle regulation and growth arrest are still largely elusive; cell cycle regulation is a complex process that ensures correct cell division and is controlled by multiple mechanisms, governed by key regulatory proteins, including the cyclin-dependent kinases (CDKs) and their regulatory inhibitors. CDKs are a family of serine/threonine protein kinases that are activated at specific points of the cell cycle and are regulated by several different mechanisms.^3^

CDK inhibitors function through inhibition of the G1 CDK cyclin complexes.^4^ Furthermore, the CDK inhibitor p21 has the ability to also inhibit DNA synthesis by binding to and inhibiting proliferating cell nuclear antigen.^5, 6, 7^ p21 protein expression is tightly regulated by the p53 tumour suppressor gene.^8^ The expression of p21 is also reported to be regulated by the recently identified Ras-associated factor 5 (NORE1A/RASSF5),^9^ a protein that is 41.8 kD and a member of the RASSF family of tumour suppressors.^10^ NORE1A expression is frequently downregulated by promoter methylation in human tumours.^11, 12^ Structurally, NORE1A contains a Ras-binding domain and was originally identified by a yeast two-hybrid screen.^13^ NORE1A can directly bind the Ras oncoprotein in a GTP-dependent manner.^14^ Exogenous expression of NORE1A has been reported to promote apoptosis by Ras-dependant and -independent mechanisms.^12^ Furthermore, NORE1A protein expression results in a decrease in the number of cells in S phase of the cell cycle,^15^ indicating that NORE1A might function as a tumour suppressor; here, we show for the first time that mCD40L but not sCD40L induces the expression of NORE1A in an NF*κ*B-dependant manner. NORE1A expression was found to contribute to mCD40L-induced cell death since inhibition of mCD40L-induced NORE1A expression resulted in reduced cell death by mCD40L. The role of CD40-induced NORE1A expression in cell cycle regulation was also examined and found to be mediated by p21 upregulation.

## Results

### Membrane-expressed but not soluble CD40L induces NORE1A expression in CD40-positive carcinomas

**Figure 1:**
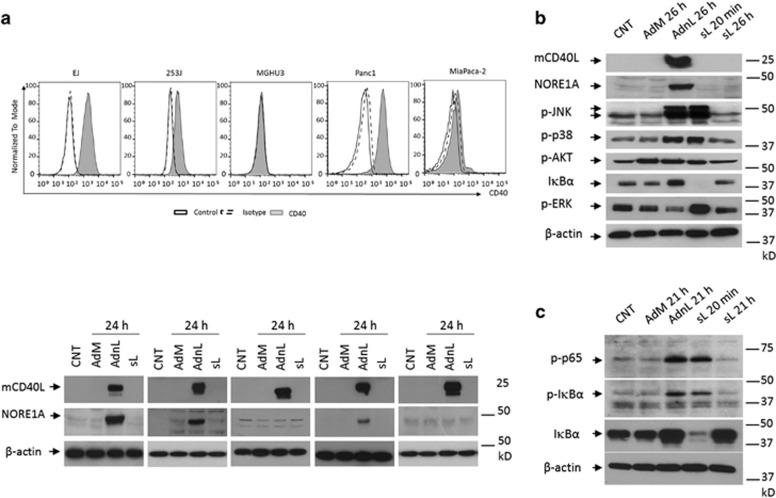
CD40 receptor activation by mCD40L but not sCD40L induces NORE1A expression in carcinomas. (**a**) The CD40-positive bladder carcinoma cell lines EJ, 253J and the CD40-negative MGHU3 bladder carcinoma cells and the CD40-positive pancreatic cell line Panc1 and the CD40-negative MiaPaca-2 cells were infected with 100 and 30 MOI of either RAdMock (AdM) or RAdnCD40L (AdnL), respectively, or left uninfected as a negative control or treated with sCD40L (sL) at a final concentration of 1 *μ*g/ml for 24 h. Cells were then examined for CD40 expression by FACS analysis and mCD40L, NORE1A expression by western blot analysis. *β*-Actin expression was examined to ensure equal loading between different samples. (**b**) EJ cells were infected with 100 MOI of either RAdMock or RAdnCD40L or left untreated as a negative control or treated with sCD40L at a final concentration of 1 *μ*g/ml for the indicated time points. Cells were lysed and examined for the expression of mCD40L, NORE1A, I*κ*B*α* and the *β*-actin as a loading control. The phosphorylated levels of JNK, AKT, p38 and ERK were also examined. (**c**) EJ cells were infected with 100 MOI of either RAdMock or RAdnCD40L or left untreated as a negative control or treated with sCD40L (1 *μ*g/ml) for the indicated time points. Total cell lysates were examined for the expression of p-p65 and p-I*κ*B*α*, I*κ*B*α* and the *β*-actin as a loading control

To examine the effect of different forms of CD40 ligation on NORE1A expression, the CD40-expressing bladder carcinoma cell lines EJ, 253J and the CD40-negative MGHU3 bladder carcinoma cells were infected with RAdnCD40L (AdnL) or treated with sCD40L at a final concentration of 1 *μ*g/ml. Cells were allowed to grow for 24 h, and NORE1A expression was examined by western blot analysis. CD40 activation by mCD40L delivered by AdnL resulted in NORE1A expression only in CD40-positive cells (Figure 1a). Interestingly, the activation of CD40 receptor by sCD40L even at high concentration (1 *μ*g/ml) failed to induce NORE1A expression in any of the examined CD40-positive carcinomas, indicating that NORE1A expression is restricted to activation of the CD40 receptor with mCD40L but not the soluble counterpart. Similarly, NORE1A expression was also detected in CD40-positive pancreatic cell line Panc1 but not the CD40-negative cell line MiaPaca-2 cells upon CD40 activation with mCD40L but not sCD40L-treated cells (Figure 1a), indicating that NORE1A induction by mCD40L is not cell-type dependent.

The finding that sCD40L failed to induce NORE1A expression in any of the examined CD40-expressing carcinomas prompted us to examine whether sCD40L retains its full biological activity and was capable of activating the CD40 receptor. Therefore, the CD40-positive EJ cells were infected with AdnL or treated with sCD40L, followed by examining of CD40 downstream signalling molecules and NORE1A expression. As shown in Figure 1b, the CD40 downstream signalling molecules were activated by CD40 ligation either by mCD40L or sCD40L evidenced by higher levels of phosphorylated JNK, p38, AKT and ERK proteins compared with untreated or RAdM-transduced control cells, however, NORE1A expression was only detected in cells transduced with AdnL but not with those treated with sCD40L. Furthermore, the undetectable level of IK*βα* in the sCD40L-treated cells indicates the activation of the NF*κ*B pathway and confirms the functional activity of sCD40L. However, the unexpected higher level of IK*βα* in AdnL-infected cells prompted us to examine the levels of phosphorylated p65 and IK*βα* in AdnL-infected cells compared with sCD40L-treated cells (Figure 1c), indeed mCD40L expression induced the phosphorylation of p65 and IK*βα* indicating NF*κ*B activation in mCD40L-expressing cells despite of accumulation of IK*βα* compared with sCD40L-treated cells. Given that mCD40L induces constitutive activation of NF*κ*B pathway compared with a transient activation by sCD40L, higher level of IK*βα* in AdnL-infected cells could be attributed to a positive feedback regulation of IK*βα* expression because of the constitutive phosphorylation of NF*κ*B subunits, where within a transient activation of NF*κ*B subunits, a rapid degradation of IK*βα* molecules is expected. Taken together, these results suggest that NORE1A expression is driven by membrane-bound but not sCD40L in CD40-positive carcinomas.

### mCD40L-induced NORE1A expression is mediated by the NF*κ*B pathway

**Figure 2:**
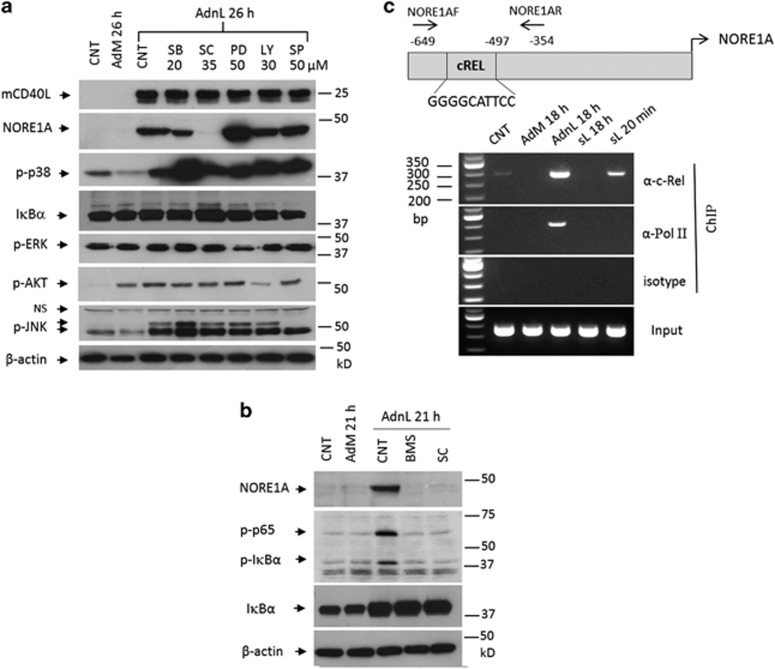
mCD40L-induced NORE1A expression is mediated by NF*κ*B activation. (**a**) EJ cells were infected with 100 MOI of either RAdMock (AdM) or RAdnCD40L (AdnL), RAdnCD40L-infected cells were either treated with p-p38 inhbitor SB203580 (SB), the NF*κ*B inhbitor SC-514 (SC), the MEK, the ERK kinase inhbitor PD98059 (PD), the AKT pathway inhbitor LY294002 (LY) and the JNK pathway inhbitor SP600125 (SP) at the following concentrations 20, 35, 50, 30, 50 *μ*M, respectively, or left untreated as control for 26 h. Cells were lysed and total protein lysates were prepared and examined for the expression of mCD40L, NORE1A, p-p38, I*κ*B*α*, p-ERK, p-AKT, p-JNK and *β*-actin as a loading control. (**b**) EJ cells were infected with 100 MOI of either AdM or AdnL or left untreated as a negative control, AdnL-infected cells were either treated with the NF*κ*B inhbitor SC-514 (SC; 35 *μ*M) or the BMS-345541 (BMS; 8 *μ*M) or left untreated as a control for 21 h. Cells were lysed and total cell lysates were examined for the expression of p-p65 and p-I*κ*B*α*, I*κ*B*α*, NORE1A and the *β*-actin as a loading control. (**c**) EJ cells were infected with 100 MOI of either AdM or AdnL or left untreated as a negative control or treated with sCD40L (1 *μ*g/ml) for the indicated time, ChIP assays were performed and one-tenth of the volume of the chromatin obtained was used for PCR as input, and the remaining volume was immunoprecipitated with anti-c-Rel (*α*-c-Rel) or anti-RNA polymerase II (*α*-Pol II) or Rabbit isotype (isotype) antibodies as described in materials and methods. Precipitated DNA spanning the NORE1A gene promoter was evaluated by PCR under the following conditions (95 °C 5 min, (93 °C 30 s, 64.3 °C 30 s, 72 °C 1 min) × 35, 72 °C 5 min), the PCR products were resolved by 2% agarose gel electrophoresis. The c-Rel-binding site within the NORE1A gene promoter and the sites where the primers for amplification of NORE1A gene promoter spanning the c-Rel-binding sequence were designed are indicated in the schematic diagram

To investigate the signalling pathway downstream of CD40 that mediates NORE1A expression by mCD40L, EJ cells were infected with AdnL and then treated with chemical inhibitors targeting p38 MAP kinase, SB203580^16^ (SB); NF*κ*B, SC-514^17^ (SC); MEK, the ERK kinase inhibitor, PD98059^18^ (PD); PI3K/Akt, LY294002^19^ (LY) or JNK, SP600125^20^ (SP) at the indicated concentrations (Figure 2a). The finding that treatment of AdnL-infected EJ cells with the NF*κ*B inhibitor SC-514 but not the other inhibitors abolishes NORE1A expression indicates that mCD40L-induced NORE1A expression is mediated by CD40-induced NF*κ*B activation. The specificity of these inhibitors was confirmed by its ability to inhibit phosphorylation of AKT, ERK and JNK without cross-interfering with other signalling molecules. However, the higher levels of phospho-p38 detected in SB-treated AdnL-infected cells is attributed to its mode of action through the inhibition of p38 MAPK catalytic activity by binding to the ATP-binding pocket, without affecting the phosphorylation of p38 MAPK by upstream kinases.^21^

Interestingly, the inhibition of ERK by the ERK inhbitor (PD) resulted in increased NORE1A expression in AdnL-infected cells; this could be attributed to the negative feedback of Ras activation by ERK inhibition, given that, NORE1A is known to form a complex with an active Ras, and inhibition of ERK could lead to accumulation of NORE1A protein (Figure 2a). Given that NF*κ*B inhibitor SC-514 was able to block the expression of NORE1A in EJ cells expressing mCD40L, we examined the effect of the NF*κ*B inhibitor, BMS-345541^22^ (BMS) on NORE1A expression in mCD40L-expressing cells. Indeed, inhibition of NF*κ*B activation by either SC-514 (35 *μ*M) or BMS-345541 (8 *μ*M) resulted in blocking phosphorylation of p65 and IK*βα* and NORE1A expression (Figure 2b) confirming that NORE1A expression in mCD40L-expressing cells is mediated by NF*κ*B activation. This result prompted us to examine whether NORE1A gene promoter contains any NF*κ*B-binding sites that would allow NORE1A expression following mCD40L-induced NF*κ*B activation. Indeed, NORE1A promoter sequence analysis utilising the web-available Match analysis software identified the NF*κ*B c-Rel-binding site (^-555^GGGGCATTCC^-546^) within the NORE1A promoter sequence. To examine whether c-Rel binds to the NORE1A gene promoter in mCD40L-expressing cells, ChIP experiments were performed. Chromatin from EJ cells infected with 100 multiplicity of infection (MOI) of either AdM or AdnL or uninfected or treated with sCD40L for 20 min or 18 h was used for immunoprecipitation with an anti-c-Rel, anti-RNA polymerase II (Pol II) or rabbit isotype antibodies and precipitated DNA spanning the NORE1A promoter c-Rel-binding site was assessed by PCR. As shown in Figure 2c, NORE1A gene prompter precipitated with cells treated with sCD40L for 20 min but not for 18 h or AdM-infected cells, the recruitment of c-Rel to NORE1A gene promoter significantly increased within mCD40L-expressing cells compared with sCD40L-treated or untreated control cells. In contrast, Pol II was only recruited to the NORE1A gene promoter in mCD40L-expressing cells but not in sCD40L-treated cells. Together, these data confirm that mCD40L-induced NORE1A expression is directly regulated by mCD40L-induced NF*κ*B activation.

### Inhibition of NORE1A expression decreases mCD40L-induced cell death

**Figure 3:**
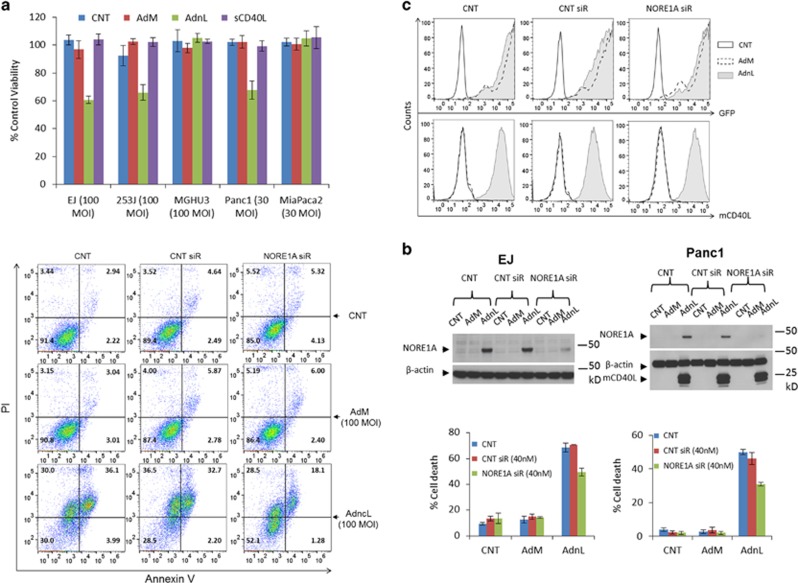
Inhibition of mCD40L-induced NORE1A expression reduces CD40L-induced cell death. (**a**) The bladder carcinoma cell lines EJ, 253J and the CD40-negative MGHU3 cells and the pancreatic cell line Panc1 and the CD40-negative MiaPaca-2 cells were infected with 100 and 30 MOI of either RAdMock (AdM) or RAdnCD40L (AdnL), respectively, or left uninfected as a negative control (CNT) or treated with recombinant sCD40L at a final concentration of 1 *μ*g/ml for 30 h. Cell viability was assessed by WST-1 assay. Results represent the mean of triplicate samples ±S.D. (**b**) EJ and Panc1 cells were transfected with 40 nM of either off-target siRNA or NORE1A siRNA or left untransfected as a negative control for 48 h. Cells were lightly trypsinzed and collected, followed by infection with 100 and 30 MOI, respectively, of either AdM or AdnL or left uninfected as a negative control for further 24 h. Cell death was then assessed by FACS analysis utilising the PI and annexin v staining method. Results represent the mean of triplicate samples ±S.D. NORE1A inhibition by NORE1A siRNA in AdnL-infected EJ or Panc1 cells was confirmed by western blot analysis, in addition to mCD40L and *β*-actin as a loading control. (**c**) Expression of mCD40L and GFP in EJ cells were examined by FACS analysis to ensure equal MOI infection between different treatments

Previously, we and others have shown that CD40 receptor activation with mCD40L but not sCD40L directly induces apoptosis,^1, 2^ also as shown in Figure 3a. NORE1A has been reported to act as a tumour suppressor that upon expression can lead to a decrease in the number of cells in S phase of the cell cycle via a G1 growth arrest.^15^ Therefore, we examined whether mCD40L-induced NORE1A expression contributes to the mCD40L-induced cell death in CD40-positive carcinomas. Thus, the CD40-positive carcinoma EJ and Panc1 cell lines were transfected with NORE1A small interfering RNA (siRNA) and then infected with AdnL. Cell death was then assessed using the annexin V and propidium iodide (PI) staining. Inhibition of NORE1A expression in AdnL-infected cells resulted in a significant reduction in the cell death induced by mCD40L. The inhibition of NORE1A expression by NORE1A siRNA transfection was confirmed and revealed a significant reduction in the level of NORE1A protein compared with the off-target siRNA or untransfected control cells (Figure 3b).

### Induction of TNF-*α* does not contribute to the mCD40L-induced cell death

**Figure 4:**
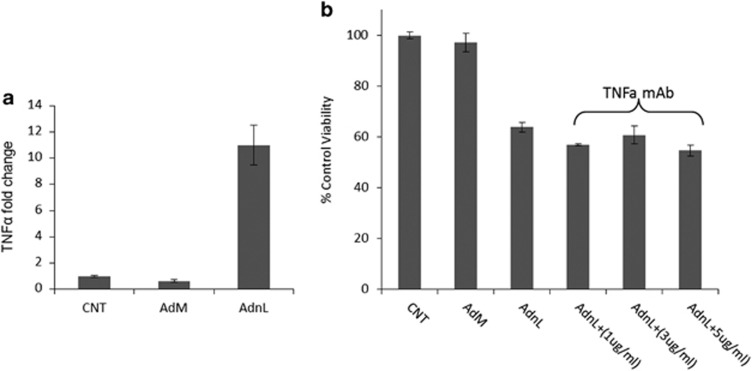
mCD40L-induced TNF*-α* does not contribute to CD40L-induced cell death. (**a**) EJ cells were infected with 100 MOI of either RAdMock (AdM) or RAdnCD40L (AdnL) or left uninfected as a negative control for 24 h, RNA was extracted utilising the EZ-RNA total isolation kit and the cDNA was prepared by reverse transcription. The expression of TNF*-α* was examined by qRT-PCR technique. Results are mean of triplicate samples ±S.D. (**b**) EJ cells were infected with 100 MOI of either RAdMock (AdM) or RAdnCD40L (AdnL) or left uninfected as a negative control and plated at a density of 6000/100 *μ*l/well in 96-well microplate. AdnL-infected cells were either treated with 1, 3 or 5 *μ*g/ml of TNF*-α* monoclonal neutralising antibody or left untreated as a control for 28 h. Cell viability was then assessed using the WST-1 assay. Results are mean of triplicate samples ±S.D.

Recent data suggest that NORE1A expression mediates TNF-*α*-induced cell death, such that inhibition of NORE1A expression results in resistance to TNF-*α*-mediated cell death.^23^ Therefore, we examined whether AdnL induces TNF-*α* expression that contributes to the observed mCD40L-induced cell death. Thus, EJ cells were infected with AdnL and the level of TNF-*α* expression was examined by quantitative PCR (qPCR) analysis. mCD40L expression resulted in 10-fold increase in the TNF-*α* mRNA expression compared with RAdM-infected or uninfected control cells (Figure 4a). To examine whether TNF-*α* contributes the AdnL-induced cell death, cells were treated with escalating doses of TNF-*α*-neutralising monoclonal antibody (1, 3 and 5 *μ*g/ml) (Clone D2H4 mAb#11969, Cell Signalling), followed by assessment of cell viability. Addition of TNF-*α*-neutralising monoclonal antibody did not rescue cells from mCD40L-induced cell death even at higher concentration, indicating that TNF-*α* does not contribute to mCD40L-induced cell death (Figure 4b).

### mCD40L induces G1 growth arrest in a NORE1A-dependant manner

**Figure 5:**
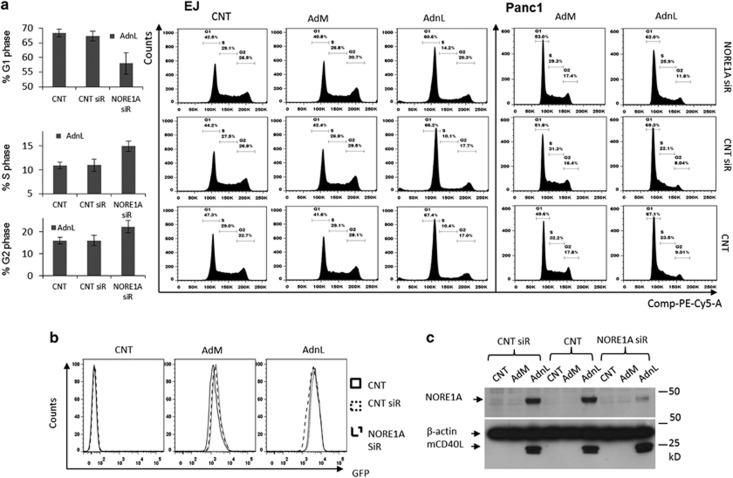
Inhibition of mCD40L-induced NORE1A expression enhances G0/G1-S phase transition. EJ and Panc1 cells were transfected with 40 nM of either off-target siRNA or NORE1A siRNA or left untransfected as a negative control for 48 h. Cells were lightly trypsinzed and collected followed by infection with 100 and 30 MOI, respectively, of either RAdMock (AdM) or RAdnCD40L (AdnL) or left uninfected as a negative control for further 18 h. (**a**) Cell cycle was analysed using the PI staining method by flow cytometery. Results represent the average of two independent experiments. (**b**) GFP expression in EJ cells was examined across all the treatments by FACS to ensure equal infection. (**c**) EJ cells were lysed *in situ* and total protein lysates were examined for NORE1A, nCD40L and *β*-actin expression

The finding that inhibition of mCD40L-induced NORE1A expression results in reduced cell death in EJ and Panc1 cells suggests that NORE1A has an anti-proliferative effect. Indeed, exogenous expression of NORE1A was reported to reduce the number of cells in S phase of the cell cycle.^15^ Therefore, we next examined the effect of mCD40L on cell cycle progression and the role of NORE1A in this process. Thus, EJ and Panc1 cells were transfected with the NORE1A siRNA and then infected with AdnL followed by cell cycle analysis. mCD40L expression in both EJ and Panc1 cells resulted in G1 growth arrest and inhibition of NORE1A expression resulted in reduced number of cells in G1 phase associated with an increase in the number of cells in S and G2 phases (Figure 5). These results clearly highlight the role of mCD40L to modulate cell cycle progression via NORE1A expression.

### mCD40L induces p21 expression in a NORE1A-mediated mechanism

**Figure 6:**
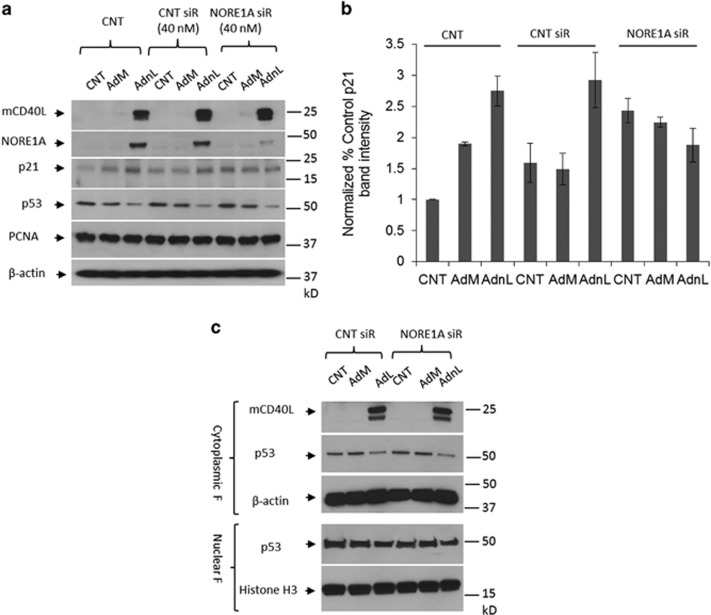
mCD40L-induced p21 expression is mediated by NORE1A protein. (**a** and **b**) EJ cells were transfected with 40 nM of either off-target siRNA or NORE1A siRNA or left untransfected as a negative control for 48 h. Cells were lightly trypsinzed and collected followed by infection with 100 MOI of either RAdMock (AdM) or RAdnCD40L (AdnL) or left uninfected as a negative control for further 24 h. Cells were lysed *in situ* and total protein lysates were examined for NORE1A, mCD40L, p21, p53, proliferating cell nuclear antigen and *β*-actin expression as a loading control (**a**). Expression levels of p21 were analysed by densitometry utilising the web-available ImageJ software and normalised to *β*-actin expression (**b**) results are average of two independent experiments ±S.D. (**c**) EJ cells were transfected with 40 nM of either off-target siRNA or NORE1A siRNA for 48 h. Cells were lightly trypsinzed and collected followed by infection with 100 MOI of either AdM or AdnL for further 24 h. Cytoplasmic and nuclear fractions were prepared and mCD40L, p53 and *β*-actin were examined in the cytoplasmic fraction, p53 and histone H3 as a loading control were examined in the nuclear fraction

We next examined whether p21 is involved in mCD40L-induced G1 growth arrest. Thus, EJ cells were transfected with the NORE1A siRNA and then infected with AdnL. Cell lysates were then examined for expression of mCD40L, NORE1A and p21. mCD40L expression resulted in the upregulation of p21 in a NORE1A-dependant manner since inhibition of NORE1A resulted in normal p21 expression compared with AdM-infected or uninfected control cells (Figures 6a and b). No significant change was detected in the expression of proliferating cell nuclear antigen, which is known to be inhibited by p21 binding. It has been reported that NORE1A expression in hepatocellular carcinomas modulates p21 expression via promoting wild-type but not mutant p53 nuclear localisation.^9^ However, in our experiment, p21 upregulation mediated by NORE1A expression (Figure 6a) appeared to be concomitant with downregulation of p53 expression in the cytoplasmic fraction, together with no evidence of increased p53 localisation in the nucleus (Figure 6c).

### Inhibition of JNK but not ERK protects cells from mCD40L-induced cell death

**Figure 7:**
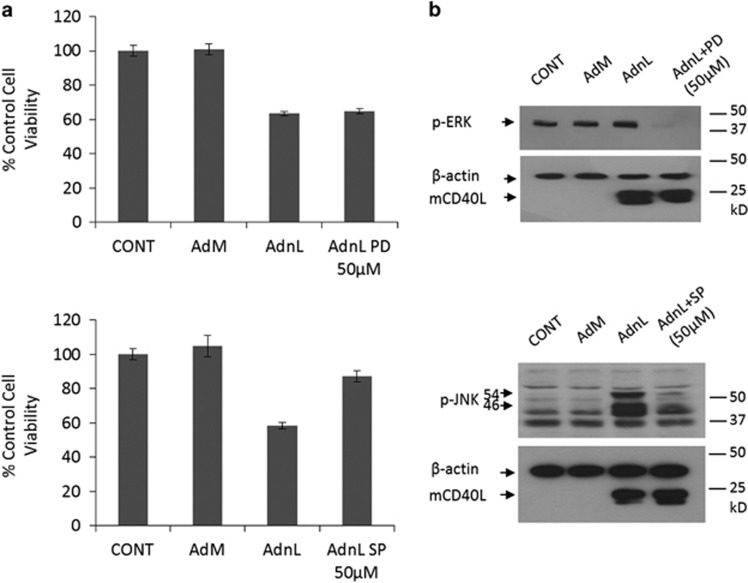
Inhibition of JNK but not ERK pathway protects cells from mCD40L-induced cell death. EJ cells were infected with 100 MOI of either RAdMock (AdM) or RAdnCD40L (AdnL) or left uninfected as a negative control, AdnL-infected cells were either cultured in a medium containing 50 *μ*M of the JNK inhibitor SP600125 or the 50 *μ*M of the ERK inhibitor PD98059 or cultured in a drug-free medium as a control for 24 h. (**a**) Cells were either assessed for cell viability by WST-1 assay reagent. Results are average of triplicate samples ±S.D. (**b**) or lysed *in situ* and total protein lysates were examined for p-JNK, p-ERK, mCD40L and *β*-actin expression as a loading control

Previously, we have shown that mCD40L-induced cell death is mediated by MAPK/JNK activation and inhibition of this pathway by pharmacological inhibitors protects cells from this effect. Given that NORE1A was identified as a Ras-binding protein,^13^ and exogenous expression of NORE1A has been reported to promote apoptosis via Ras-dependant and -independent mechanisms,^12^ we examined the effect of inhibition of the MAPK kinases on mCD40L-induced growth inhibition. Thus, EJ cells were infected with AdnL and were cultured in a medium containing the ERK inhibitor (PD98059) or the JNK inhibitor (SP600125), followed by cell viability assessment. The JNK inhibitor but not the ERK inhibitor was able to inhibit mCD40L-induced cell death, suggesting that the effects of NORE1A in context of mCD40L signalling are Ras-independent (Figure 7a). The inhibitory effect of these inhibitors on their specific targets was confirmed by western blot (Figure 7b).

## Discussion

Much research has been carried out on CD40 signalling and its ability to induce either pro-survival or pro-apoptotic signals depending on the way the CD40L engages with its receptor in carcinomas. We have shown previously that mCD40L delivered by a RAd vector induces cell death in CD40-expressing carcinomas via a mechanism involving downregulation of pro-survival signals, mainly the PI3K/AKT pathway and the sustained activation of the JNK pathway, leading to caspase activation and subsequently apoptotic cell death.

Given the complexity of mCD40L-induced growth-inhibitory effects, it is not surprising that CD40 has a role in cell cycle regulation. Here, we show for the first time that mCD40L but not sCD40L induces NORE1A expression in CD40-expressing carcinomas, a protein that has been recently identified as a Ras-binding partner and upon ectopic expression can promote cell death via Ras-dependant and -independent mechanism.^12^ The lack of NORE1A expression in sCD40L-treated cells was not due to inability of sCD40L to activate the CD40 receptor because the downstream signalling targets were activated, suggesting that NORE1A induction is restricted to CD40 activation by mCD40L.

Furthermore, the ability of the NF*κ*B inhibitors (SC-514 and BMS-345541) but not inhibitors of p38 MAP kinase, ERK, PI3K/Akt or JNK to abolish mCD40L-induced NORE1A expression, indicates that mCD40L-induced NORE1A expression is mediated by mCD40L-induced NF*κ*B activation. This was further supported by the finding of an NF*κ*B c-Rel-binding site (^−555^GGGGCATTCC^−546^) within the NORE1A promoter sequence as revealed by NORE1A promoter sequence analysis. Furthermore, the finding that c-Rel binds to the c-Rel-binding site within the NORE1A gene promoter significantly higher in mCD40L-expressing cells compared with sCD40L-treated cells for 20 min, was concomitant with RNA polymerase II recruitment to the NORE1A promoter in mCD40L-expressing but not sCD40L-treated cells either at 20 min or 18 h, indicates that sustained activation of NF*κ*B pathways is required for NORE1A expression; such a condition is only fulfilled in mCD40L-expressing cells.

Given the cell growth-inhibitory effect of mCD40L on CD40-positive carcinomas, we sought to investigate the contribution of NORE1A to these effects. This has revealed that mCD40L-induced cell death was in part through the induction of NORE1A expression because inhibition of mCD40L-induced NORE1A by specific siRNA resulted in a significant reduction in mCD40L-induced cell death compared with the off-target siRNA control. Moreover, inhibition of mCD40L-induced TNF-*α* by neutralising antibody did not rescue cells from the mCD40L-induced anti-proliferative effect. This is particularly important since NORE1A expression has been reported to sensitise cells to TNF-*α*-induced cell death.^17^ This finding clearly highlights the involvement of NORE1A in mCD40L-induced cell death. Interestingly, NORE1A was not only involved in mCD40L-induced cell death but was also involved in mCD40L-induced G1 growth arrest, such as inhibition of NORE1A expression resulted in enhancing the transition of cells from G1 to S phase.

The effect of mCD40L-induced NORE1A expression on cell cycle regulation appears to be mediated by modulation of p21 levels because NORE1A-expressing cells exhibited higher level of p21 expression compared with those targeted with specific NORE1A siRNA. p21 upregulation in mCD40L-expressing cells appears to be independent of p53 since p21 upregulation was concomitant with downregulation of p53 expression with no evidence of increased p53 localisation in the nucleus. The inability of the MEK, the ERK kinase inhibitor PD98059 to protect cells from the mCD40L-mediated growth-inhibitory effect suggests that the effect of NORE1A in this context is Ras-independent. It is likely that mCD40L-induced NORE1A expression functions in parallel with sustained JNK activation to mediate cell death given that inhibition of JNK did not affect the NORE1A expression and that NORE1A inhibition by the NF*κ*B SC-514 inhbitor did not affect the level of JNK phosphorylation (as shown in Figure 2a), however, this is currently under investigation. This study provides a further understanding of the differential effects of membrane-expressed and soluble CD40L in the context of CD40-expressing carcinomas. It also emphasises the importance of utilising membrane-expressed ligand to fully appreciate the biological effects of CD40 ligation, since this more closely resembles the physiological form in which CD40L is presented *in vivo*.

## Materials and Methods

### Maintenance of cell lines

Bladder carcinoma EJ, 253J and MGHU3 cell lines and the pancreatic cell lines Panc1 and MiaPaca-2 cells were maintained in either RPMI 1640 or DMEM supplemented with 2 mM glutamine, 10% FCS.

### Recombinant adenovirus vectors and cell infection

The replication-deficient E1, E3-deleted recombinant adenoviruses expressing either membrane-bound, noncleavable CD40L (RAdncCD40L) or GFP control (RAdMock) were constructed using methods as described.^24^ Viruses were purified by caesium chloride banding and dialyzed against a buffer containing 10 mM Tris-HCl (pH 8.0), 2 nM MgCl and 5% sucrose. Virus titres were determined using the 50% tissue culture-infective dose method, based on the development of cytopathic effects in 293 cells using serial dilutions to estimate adenovirus stock titer. Cells were infected in 10% FCS DMEM with the appropriate MOI for 2 h at 37 °C. For cell viability, infected cells were seeded in 10% FCS DMEM at 4000 cells/well in a 96-well plate or 3 × 10^5^cells/30-mm dish for western blotting analysis. The resistance of the mCD40L mutant expressed and delivered by RAdnCD40L to cleavage from the cell membrane was confirmed and described previously.^2^

### Analysis of cell surface CD40 and CD40L expression

For CD40 expression, cells were plated at 5 × 10^5^ cells/well in six-well plates for 24 h and then washed with ice-cold PBS buffer and incubated on ice for 1 h with 100 *μ*l of diluted mouse anti-CD40 Ab or mouse isotype Ab or left without treatment as unstained control. Cells were then washed three times with 100 *μ*l ice-cold PBS followed by incubation with the anti-mouse IgG APC Ab for 1 h. Following three washes with 100 *μ*l ice-cold PBS, cells were fixed in 1% paraformaldehyde and analysed by flow cytometry.

For CD40L expression, cells were infected with RAdncCD40L at an appropriate MOI for 24 h. A total of 3 × 10^5^ cells were washed three times with ice-cold PBS buffer and incubated on ice for 45 min with 100 *μ*l of diluted mouse anti-CD40L–APC conjugate Ab or mouse isotype–APC Ab conjugate or left without treatment. Cells were then washed three times with ice-cold PBS buffer, fixed in 500 *μ*l 1% paraformaldehyde and analysed by flow cytometry.

### Pharmacological inhibitors

The JNK inhibitor (SP600125) was from Alexis Biochemicals (Plymouth Meeting, PA, USA). The PI3K inhibitor (LY294002) and the IKK2 inhibitor (SC-514 and BMS-345541) were from Calbiochem (Nottingham, UK). The ERK inhibitor (PD98059) and the p38 MAP kinase inhibitor (SB203580) were from Cell Signaling Biotechnology (Beverly, MA, USA). Inhibitors were reconstituted in DMSO (Sigma-Aldrich, Gillingham, Dorest, UK).

### Quantitative real-time polymerase chain reaction

RNA was extracted using the EZ-RNA total isolation kit (Biological Industries, Kibbutz Beit Haemek, Israel), and cDNA was synthesised from 2 *μ*g of total RNA utilising the RETROscript RNase reverse transcription kit (Ambion Europe, Huntingdon, UK) according to the manufacturer's instructions. Real-time PCR (qPCR) was then performed for TNF-*α* utilising the TNF-*α* and GAPDH QuantiTect Primer Assay kits (cat TNF-*α* QT01079561, GAPDH QT00079247, Qiagen, Manchester, UK), LightCycler PCR was performed with DNA SYBR Green I according to the manufacturer's instructions (Roche Diagnostics, West Sussex, UK). TNF-*α* mRNA level was normalised to GAPDH mRNA to determine relative expression ratios.

### RNA interference studies

siRNA directed to NORE1A (J-010585-07-0010, Dharmacon, CO, USA), p21 (SC-29427) and a control siRNA-A (SC-37007), which does not lead to specific degradation of any known cellular mRNA, were from Santa Cruz Biotechnology (Santa Cruz, CA, USA). Cells were transfected with 40 nM of the siRNA utilising the DHARMAFECT 1 transfection reagent (T-2001-02, Dharmacon) according to the manufacturer's instructions.

### Annexin V staining of apoptotic cells

Cells were transfected with 40 nM of either off-target siRNA or the target siRNA or left untransfected as a negative control for 48 h. Cells were lightly trypsinzed and collected, followed by infection with the appropriate MOI of either RAdMock (AdM) or RAdnCD40L (AdnL) or left uninfected as a negative control for further 24 h. Cells were harvested and analysed with Annexin V Apoptosis Detection Kit APC (88-8007, eBioscience, San Diego, CA, USA) according to the manufacturer's instruction (eBioscience). Cells were harvested, and the percentage of annexin V-APC-positive cells was determined within 2 × 10^4^ cells of the population by flow cytometry.

### Antibodies and immunoblotting

Phosphospecific JNK, AKT, p38, p65, I*κ*B*α* and ERK antibodies and TNF*-α*-neutralising antibody were all purchased from Cell Signaling Technology. NORE1A monoclonal antibody, CD40L, p21, p53, c-Rel, RNA polymerase II, rabbit isotype and histone H3 antibodies were all from Santa Cruz Biotechnology. CD40L–APC and isotype–APC conjugates were from eBioscience. For immunoblotting, 10–50 mg protein was separated by SDS-PAGE, transferred onto polyvinylidene difluoride membrane (for phosphoproteins) or Biotrace nitrocellulose membranes (Pall, Port Washington, NY, USA) (for nonphosphoproteins), and blocked with 10% of BSA (for phosphoproteins) or nonfat milk (for nonphosphoproteins) dissolved in PBS supplemented with 0.1% Tween 20 for 1 h. After three washes with PBS supplemented with 0.1% Tween 20, membranes were incubated overnight at 4 °C with primary antibody and for 1 h at room temperature with the appropriate secondary antibody followed by ECL (Amersham Biosciences, Piscataway, NJ, USA). Cytoplasmic and nuclear extracts from EJ cell were prepared by using the NE-PER nuclear and cytoplasmic extraction kit (Thermo Scientific, MA, USA, cat. 78835) according to the manufacturer's instructions.

### Chromatin immunoprecipitation (ChIP) assay

EJ cells were infected with 100 MOI RAdMock or RAdnCD40L or left uninfected for 18 h or treated with sCD40L (1 *μ*g/ml) for 20 min or 18 h, followed by cross-linking by addition of 37% formaldehyde directly into the cultured media to a final concentration of 1% for 10 min at room temperature. To abort the cross-linking, glycine was added to a final concentration of 0.137 M. Cells were then washed with cold PBS and harvested in PBS with 0.5% NP-40 and 0.5 *μ*M phenylmethylsulfonyl fluoride. Following centrifugation at 1000 r.p.m. for 5 min at 4 °C, cells were resuspended in 1 ml swelling buffer (25 mM HEPES, pH 7.8, 1.5 mM MgCl2, 10 mM KCl, 0.5% NP-40, 1 mM DTT, 0.5 *μ*M phenylmethylsulfonyl fluoride, protease inhbitor) and incubated for 10 min on ice. For the nuclei extraction, cells were homogenised by around 20 strokes using Dounce homogeniser and the nuclei were collected by centrifugation at 5000 r.p.m., the nuclei were resuspended in 300 *μ*l sonication buffer (50 mM HEPES, pH 7.9, 140 mM NaCl, 1 mM EDTA, 1% Triton X-100, 0.1% sodium deoxycholate, 0.1% SDS and protease inhibitors), and sonicated on ice using the Branson Sonifier 250 (Branson Ultrasonics, Shanghai) to obtain average chromatin fragments between 200 and 1000 bp. The samples were then cleared by centrifugation at 14 000 r.p.m. for 20 min at 4 °C and the supernatant was collected and topped up with sonication buffer to 1 ml. Following preclearing by incubation with 40 *μ*l of protein G-Sepharose beads while rotating at 4 °C for 2 h, samples were spun at 4000 r.p.m. for 3 min at 4 °C and 100 *μ*l of the precleared supernatant was used as input and the remaining volume was immunoprecipitated with 5 *μ*g of anti-c-Rel, anti-RNA polymerase II or rabbit isotype antibodies overnight at 4 °C. Immunocomplexes were captured by incubating with protein G-Sepharose beads for 2 h at 4 °C, and the beads were washed twice with each of the following buffers, sonication buffer, sonication buffer with high salt (500 mM NaCl), buffer containing 20 mM Tris-Cl (pH 8), 1 mM EDTA, 250 mM LiCl, 0.5% sodium deoxycholate, protease inhbitor and once with TE (10 mM Tris-Cl (pH 8), 1 mM EDTA, 0.5 mM phenylmethylsulfonyl fluoride and protease inhbitor) by incubation on a rotating platform for 3 min at 4 °C. The immunocomplexes were eluted from the protein G-Sepharose beads by incubating with 200 *μ*l elution buffer (50 mM Tris (pH 8.0), 1 mM EDTA, and 1% SDS) at 65 °C for 10 min. To reverse the cross-links, the samples including the input samples were adjusted to contain 200 mM NaCl and incubated at 65 °C for 5 h, followed by addition of proteinase K and incubation for 2 h at 55 °C. The DNA was then extracted with phenol-chloroform and precipitated with ethanol. Precipitated chromatin was examined for NORE1A promoter immuneprecipitation by PCR utilising the NORE1A forward 5'-GAAAGAGGAATACCCTACCCGGC-3' and reverse 5'-TGAGTAGGGCCCCTCCGTCTA-3' primers that correspond to the NORE1A promoter region (−650 to −354 bp) for 35 cycles. The products of the PCR amplifications were resolved by 2% agarose gel electrophoresis.

### Cell cycle analysis

Cell cycle was analysed using PI staining. Briefly, cells were harvested by light trypsinization, following two washes with 2 ml ice-cold PBS, cells were pelleted by centrifugation at 1000 r.p.m. and fixed by resuspension in ice-cold 70% ethanol for 2 h on ice. Following fixation, cells were washed with 2 ml ice-cold PBS, then resuspended in 50 *μ*l of Ribonuclease A (100 *μ*g/ml PBS) and incubated at 37 °C for 20 min, followed by addition of 300 *μ*l PI (50 *μ*g/ml PBS)/10^6^ cells. Cells were allowed to stain with the PI for 30 min at room temperature, followed by analysis using flow cytometry.

## Abbreviations

MOI: multiplicity of infection
mCD40L: membrane-bound CD40L
RAdncCD40L: recombinant adenovirus expressing membrane-bound, noncleavable CD40L
RAdMock: recombinant adenovirus expressing GFP control
rsCD40L: recombinant soluble CD40L
siRNA: small interfering RNA
CHIP: Chromatin immunoprecipitation
